# Novel Kinin B_1_ Receptor Splice Variant and 5′UTR Regulatory Elements Are Responsible for Cell Specific B_1_ Receptor Expression

**DOI:** 10.1371/journal.pone.0087175

**Published:** 2014-01-27

**Authors:** Faang Y. Cheah, Svetlana Baltic, Suzanna E. L. Temple, Kanti Bhoola, Philip J Thompson

**Affiliations:** 1 Molecular Genetics and Inflammation Unit, Lung Institute of Western Australia (LIWA), Perth, Western Australia, Australia; 2 Centre for Asthma, Allergy and Respiratory Research (CAARR), School of Medicine and Pharmacology, University of Western Australia, Perth, Western Australia, Australia; 3 Department of Respiratory Medicine, Sir Charles Gairdner Hospital, Western Australia, Perth, Western Australia, Australia; 4 Department of Cellular Pathology, Ausiral University of Chile, Valdivia, Chile; International Centre for Genetic Engineering and Biotechnology, Italy

## Abstract

The kinin B_1_ receptor (B_1_R) is rapidly upregulated after tissue trauma or inflammation and is involved in cancer and inflammatory diseases such as asthma. However, the role of the: promoter; a postulated alternative promoter; and spliced variants in airway epithelial and other lung cells are poorly understood.

We identified, in various lung cell lines and leucocytes, a novel, naturally occurring splice variant (SV) of human B_1_R gene with a shorter 5′untranslated region. This novel SV is ≈35% less stable than the wild-type (WT) transcript in lung adenocarcinoma cells (H2126), but does not influence translation efficiency. Cell-specific differences in splice variant expression were observed post des[Arg10]-kallidin stimulation with delayed upregulation of SV compared to WT suggesting potentially different regulatory responses to inflammation. Although an alternative promoter was not identified in our cell-lines, several cell-specific regulatory elements within the postulated alternative promoter region (negative response element (NRE) −1020 to −766 bp in H2126; positive response element (PRE) −766 to −410 bp in 16HBE; −410 to +1 region acts as a PRE in H2126 and NRE in 16HBE cells) were found.

These findings reveal complex regulation of B_1_R receptor expression in pulmonary cells which may allow future therapeutic manipulation in chronic pulmonary inflammation and cancer.

## Introduction

Kinins (bradykinin, des[Arg^9^]-BK, and des[Arg^10^]-kallidin (DAKD)) are biologically active peptides formed by the enzymatic action of the classical tissue (KLK1) and plasma (KLKB1) kallikreins on endogenous protein substrates called H- and L- kininogens. Kinins are primarily pro-inflammatory and can affect processes such as cell proliferation and migration [1]. These effects are mediated through two G-protein coupled kinin receptors, one of which is the kinin B_1_ receptor (B_1_R) [2], [3]. Kinin B_1_ receptor is usually latent under normal physiological conditions but is quickly upregulated following initiation of inflammatory pathways [4]–[7].

Airway epithelial cells not only provide a protective lining but also initiate and regulate airway inflammation and tissue repair. The airways are constantly exposed to both exogenous and endogenous stimuli including antigens, particulates, chemicals, mediators and pathogens. Epithelial cell stimulation frequently results in inflammation which following repeated insults may lead to cell death, fibrosis and epigenetic changes that may favour tumorigenesis [8]. It has been suggested that B_1_R plays a role in sustaining and amplifying chronic inflammation [9]. It is expressed in pulmonary epithelial cells including human bronchial epithelial cells (16HBE and BEAS-2B) and human lung adenocarcinoma (A549) cell lines [9], [10]. Furthermore, B_1_R expression is induced in inflammation and tissue injury [11], [12]. Thus, tobacco smoke increases expression of B_1_R expression in rat trachea [13], leading to increased airway hyperresponsiveness [14]. B_1_R is also involved in bronchial hyperresponsiveness in rodent models of asthma [15], [16]. In humans, B_1_R expression is increased in eosinophils from asthmatic patients [17] and in nasal tissue of patients with allergic rhinitis after allergen challenge which was not observed in healthy subjects [9]. Dexamethasone, however, reduced basal expression of B_1_R and suppressed its upregulation by proinflammatory stimuli [18]. These studies all suggest that B_1_R expression is involved in the pathogenesis of chronic inflammation in allergic and smoke-related diseases such as asthma, lung cancer and COPD.

Despite its significant importance, the regulation of B_1_R expression is not clear. Human B_1_R gene contains three exons, with the first and the second being non-coding. Characterisation of the 5′ flanking core promoter region has shown the presence of a functional TATA-box and other regulatory elements that are cell-specific [19]. A positive regulatory element (PRE) functioning as an enhancer has been identified at −604 to −448 bp while a negative regulatory element (NRE) that ablates the enhancer activity is identified at −682 bp to −604 bp region relative to the transcription start site (TSS) [19], [20]. Detailed footprint analysis of the promoter region suggests possible binding by several transcription factors such as GATA-1, PEA3, AP-1, CAAT, Sp1, Pit-1a, Oct-1 and CREB [21].

It has been suggested there is a second, alternative promoter, located in intron II, as well as additional regulatory elements [20], [22], [23]. This region demonstrates cell specific activity [18]. While this region shows stronger basal promoter activity than the core promoter in HepG2 cells, it exhibits properties of a weaker promoter in vascular smooth muscle cells [18]. Whether this region functions as a promoter, particularly in inflammation as an inducible promoter, is still debated. So far, a single TSS in the kinin B1 receptor has been identified supporting the presence of only a single core promoter [21], [23].

In the current study we investigated the existence and function of this putative B_1_R alternative promoter in human pulmonary cells. While no additional TSS was found a novel 5′UTR splice variant (SV) was identified. The expression and function of the novel B_1_R SV and wild-type WT along with the role of 5′UTR regulatory elements was investigated further in a variety of lung cells. Our findings reveal that a novel B1R splice variant and promoter regulatory elements determine tissue-specific B1R expression.

## Methods

### Culturing human airway immortalised cell-lines

16HBE, A549, NHLF, HFLF, H520 and H2126 (Table 1) cells were obtained from the American Type Culture Collection (Rockville, MD). 16HBE, NHLF and HFLF were cultured in complete growth media comprised of Dulbecco's modified Eagle's medium (DMEM; Invitrogen) containing 4 mM L-glutamine (Invitrogen), while A549, H520 and H2126 cells were cultured in RPMI 1640 (Invitrogen). All cell-lines were supplemented with 10% heat inactivated fetal bovine serum (GIBCO Invitrogen), 100 IU/ml penicillin and100 µg/ml streptomycin. The cells were maintained in a humidified atmosphere in 5% CO_2_ at 37°C and were subcultured by incubating with 0.05% trypsin-0.5 mM ethylenediaminetetraacetate (Invitrogen) at a ratio of 1∶3 – 1∶4, weekly.

**Table 1:** Lung cell lines screened for B_1_R mRNA expression,

Cell lines	Description
HFLF	Human fetal lung fibroblasts
NHLF	Adult human lung fibroblasts
H2126	Human lung adenocarcinoma
A549	Human lung adenocarcinoma
16HBE	SV-40 transformed normal human bronchial epithelial
H520	Human lung squamous cell carcinoma

For cell stimulation purposes, cells were incubated in serum-free media (Invitrogen) as serum has been shown to stimulate B_1_R expression. Normal cell culture media was replaced with serum-free media for 12 hr prior to the start of stimulation, the cells were then washed once with 1X PBS before being incubated in the absence and presence of the B_1_R agonist desArg^10^KD (DAKD)(Sigma Aldrich) at 100 nM and 1 µM or lipopolysaccharide (LPS)(Sigma Aldrich) at 0.1 µg/µl, for 3, 6 and 24 hr.

### PCR conditions

PCR amplification reactions were carried out in a reaction mix containing 1X PCR buffer, 1.5 mM – 2.5 mM MgCl_2_, 5 µM of each dATP, dGTP, dCTP and dTTP (Promega, Madison, WI), 10 pmoles of each forward and reverse primer (Invitrogen or GeneWorks) and 1 U of Taq polymerase (Qiagen). For each PCR reaction, 30 ng-100 ng of DNA was used as a template and the reaction was made up to 25 uL with PCR grade water (Fisher Biotech). PCR cycling conditions were as follows: initial denaturation at 94°C for 3 min; 35 to 40 cycles of product amplification at 94°C for 30, 58–65°C for 30 s, 72°C for 30–60 s; final extension at 72°C for 5 min and finally, temperature hold at 4°C.

### Reverse Transcription- Polymerase Chain Reaction (RT-PCR)

Total RNA was extracted from l6HBE and H2126 cells using RNeasy mini kits (QIAGEN) as described by the manufacturer and quality confirmed with sharp 28S and 18S ribosomal bands on denaturing agarose gel electrophoresis with ethidium bromide staining. Single-stranded cDNA was generated using Omniscript reverse transcriptase (QIAGEN) in a 20-µl reaction mixture containing reaction buffer (50 mM Tris-HCl, pH 8.3, 75 mM KCl, 3 mM MgCl_2_, 10 mM dithiothreitol), 0.5 mM dNTP, 0.5 µg oligo(dT)(Invitrogen), 10 U rRNasin (Promega, Madison, WI), and 2 µg of total RNA. The reaction was incubated for 1 h at 37°C.

Amplification of cDNA by PCR was performed using oligonucleotide primer pairs (GeneWorks, Australia) for the human B1 receptor and the internal control superoxide dismutase 1 (SOD1;Table 2). SOD1 was used as an internal control as its expression was consistent under different stimulation conditions as verified prior to commencing real-time PCR. The reactions were performed in a 25-µl reaction mixture containing reaction buffer (20 mM Tris-HCl, pH 8.4, 50 mM KCl, 2.5 mM MgCl_2_), 0.2 mM dNTP, 2.5 U Taq polymerase (QIAGEN), and 1–2 µl of cDNA. Each primer was added at a final concentration of 0.2 µM. PCR was for 30 to 35 cycles, each cycle consisting of 30 s denaturation at 94°C, annealing at 60°C for 20 s, and extension at 72°C for 50 s. PCR reaction products were separated on 2% agarose gels containing 50 µg/ml ethidium bromide and visualized under UV light.

**Table 2:** Primers used in this study.

Primer	Description	Sequence (5′ → 3′)
B1R Fow2A	Forward primer for B1R detection in RT-PCR	CCCAACTACAGTTGTGAACGC
B1R Rev 1	Reverse primer for B1R detection in RT-PCR	CCAGGTAGATTTCTGCCACG
B1R Rev	Reverse primer for B1R detection in RT-PCR	GGGGGAGATGGTAGCTGAAT
B1R WT F	Forward primer for B1R WT detection in RT-qPCR	TTGCTGGGACCACAGGTCACT
B1R SV F	Forward primer for B1R SV detection in RT-qPCR	CATTTTCTGCCTGAGTCACT
B1R Rev qPCR	Reverse primer for B1R WT and SV detection in RT-qPCR	GCTTCTGGAGCATTGTCACAG
Sod1 Fow	Forward primer for SOD1 detection in RT-PCR	GAGAGGCATGTTGGAGACTTG
Sod1 Rev	Reverse primer for SOD1 detection in RT-PCR	TTCATGGACCACCAGTGTGC
BglII Fow	Forward primer for E2I2-Luc cloning	ACAGATCTGTCTCAGTCCGTCGGCCCAGACT
HindIII Rev	Reverse primer for E2I2-Luc cloning	GCTAAGCTTCCTGAAATGAACAGAAGG
B1R Fow	Forward primer for B1R detection in RT-PCR	GCCTCTTTCAGGTCACTGTGC
MluI Fow B1R core	Forward primer for CP-Luc and CP-E2I2-Luc cloning	TCCGAAGTCCAGCTCACTCA
NheI Rev B1R core	Forward primer for CP-Luc and CP-E2I2-Luc cloning	GCTAGCTCAGGCAGAAAATGAAGGCGT
B1R HindIII -21R	Reverse primer for E2I2 Δ410-Luc, E2I2 Δ766-Luc, CP-E2I2 Δ410-Luc and CP-E2I2 Δ766-Luc cloning	ACTGAAGCTTGCACAGTGACCTGAAATGAAC
B1R XhoI -766F	Forward primer for E2I2 Δ766-Luc and CP-E2I2 Δ766-Luc cloning	AATCTCGAGCACACCCGGCCAATACTCATGTT
B1R XhoI -410F	Forward primer for E2I2 Δ410-Luc and CP-E2I2 Δ410-Luc cloning	AATCTCGAGCCTGTAGATCCTGACAACAGCC
GR 5′Primer	Primers supplied from Invitrogen for 5′ RACE	GGACACTGACATGGACTGAAGGAGTA
GR 5′Nested	PCR that binds to the RNA oligo	ACTGACATGGACTGAAGGAGTA
RT Rev 3	Reverse primer for 5′ RACE PCR	TTGATGACCCCGTTGATGACAC
RT Rev 2	Reverse nested primer for 5′ RACE PCR	CAGATATTCTCTGCCCAGAAGG

### Real-time PCR

Reactions for real-time PCR contained 1× Platinum SYBR Green qPCR SuperMix-UDG (Invitrogen) and 100 nM of each primer. The PCR conditions were 50°C for 2 min, 95°C for 2 min followed by 40 cycles of 95°C for 15 s and 60°C for 30 s. Melting curves were generated after amplification. Data were collected using the iQ5 real-time PCR machine (BioRad). Each sample was tested in duplicate. The standard curve with serial dilutions of cDNA of known concentration was used in each qPCR assay to accurately determine the expression of splice variants, while SOD1 was used for normalisation.

### Identification of the B_1_R transcription start site/s -5′

Rapid Amplification of cDNA Ends (5′-RACE) PCR was performed on 5 µg of total RNA isolated from H2126 cells using the Generacer™ kit (Invitrogen). Briefly, cDNA prepared using the Generacer™ kit (Invitrogen) was amplified using a B_1_R gene specific reverse primer RT Rev 3, and GR 5′Primer, a forward primer that anneals to ligated Generacer™RNA oligo. Nested PCR was performed using GR5′nested and RTRev2 primers (conditions as above). PCR products were subsequently analysed using gel electrophoresis before cloning into Zero Blunt TOPO PCR cloning vector (Invitrogen) using a Zero Blunt TOPO PCR Cloning Kit for Sequencing (Invitrogen) according to the manufacturer's protocol. The TOPO cloning reaction (4 µl PCR product, 1 µl salt solution and 1 µl of TOPO vector) was mixed and incubated for 5 min at RT and used for transformation of TOP10 *E. coli* chemically competent cells (Invitrogen) using heat-shock transformation. Transformed cells were plated on Luria Bertani (LB) agar plates containing ampicillin (50 µg/mL).

Single colonies were inoculated and grown overnight in LB media containing 50 µg/mL ampicillin at 37°C and plasmid DNA was extracted using Plasmid-Mini kit (Qiagen) following manufacturer's instructions. Plasmid DNA containing 5′ RACE nested PCR products were sequenced, using B_1_R primers (Table 2) to identify the 5′end/s of B_1_R.

### Construction of B_1_R regulatory region reporter plasmids

The exon 2-intron 2 (E2I2-Luc), the 5′ core promoter (CP-Luc) and the combined 5′ promoter and exon 2-intron 2 (CP- E2I2-Luc) were constructed using the forward and reverse primers detailed in Table 2. The PCR products were then digested with either BglII/HindIII (E2I2-Luc) or MluI/NheI (CP- E2I2-Luc and CP-Luc) and cloned into their respective sites of the pGL3 Basic vector (Promega). To generate the deletion constructs, various regions of the B_1_R E2I2 were amplified using E2I2-Luc as a template (primers shown in Table 2). All of the PCR fragments were flanked by an *Xho*I site at one end and *Hin*dIII site at the other end. Following *Xho*I/*Hin*dIII digestion, fragments were introduced into the corresponding restriction sites of the E2I2-Luc and CP-Luc vectors.

### Construction and transformation of wild-type and splice variant B_1_R 5′UTR

pGL3 Control vector (Promega) was digested with *HindIII and NarI* restriction enzymes and the vector backbone was gel extracted using the QIAquick Gel extraction kit (Qiagen, Valencia, CA). B_1_R WT and SV 5′UTR were amplified using primers that contains *Hin*dIII and *NarI* restriction sites in its 5′-ends (Table 2). The PCR cycling conditions were 94°C for 3 min, followed by 35 cycles at 94°C for 30 s, 65°C for 20 s, and 72°C for 50 s. The final extension step was at 72°C for 5 min. The PCR products were purified and digested with the HindIII and NarI restriction enzymes and ligated with the pGL3 Control backbone with T4 DNA ligase at 4°C overnight. Transformation into the *Escherichia coli* strain JM109 was performed by heat shock. Purification of plasmid was performed with QIAprep spin miniprep kit (Qiagen). Constructs were sequenced in the forward and reverse direction to confirm product fidelity.

### Sequence analysis

Sequences were aligned and analysed using the program Clustal W (http://align.genome.jp/).

*In silico* sequence analysis of putative transcription factor binding sites in the B_1_R alternative promoter (1020 bp upstream of B_1_R exon III) was performed using Transcription Factor Search (Copyright 1994-2000, Yutaka Akiyama).

### Transient Transfections and Luciferase Assays

Transfections of 16HBE and H2126 were performed in 96-well tissue culture plates at a 70% confluence using Lipofectamine 2000 (Invitrogen). pRL-TK (Promega, Madison, WI) was used as a control for measuring transfection efficiency. The transfection mixture was replaced with fresh media (DMEM for 16HBE and RPMI-1640 for H2126) 6 h later. Forty-eight hours after transfection, the cells were harvested and the dual-luciferase reporter assay system (Promega) was used following manufacturer's instructions. The cells were lysed in passive lysis buffer (Promega), incubated at room temperature for 5 min and then transferred immediately to −80°C until required. The firefly luciferase emission and Renilla luciferase activity were measured in cell extract (20 µl) in an opaque 96-well plate using a luminometer (Perkin Elmer). The ratio of firefly luciferase activity to Renilla luciferase activity was calculated from each well to obtain relative luciferase activity (RLU).

### mRNA stability

Actinomycin D (ActD) was used as a highly specific inhibitor of the formation of new RNA. In this experiment, 5 µg/ml Act D was added to 16HBE and H2126 cells subcultured in 6-well plates 24 h after seeding (90% confluency). The cells were harvested at 0, 1, 3 and 5 hr after treatment with Act D, and total RNA extracted using RNeasy mini kits (Qiagen). The quantity of transcripts was determined using transcript-specific real-time PCR primers (Table 1). The use of a maximum time of 5 hrs was based on previous studies where total B_1_R mRNA half-life was documented to be well under 5 h [7], [24].

### Statistical analysis

Statistical analyses were performed using one-way ANOVA followed by a Tukey's post-test or by Student's t-test (GraphPad Prism 5.0). P<0.05 were considered to be statistically significant.

## Results

### Human lung cell lines express varying levels of basal kinin B_1_ receptor mRNA

**Figure 1:**
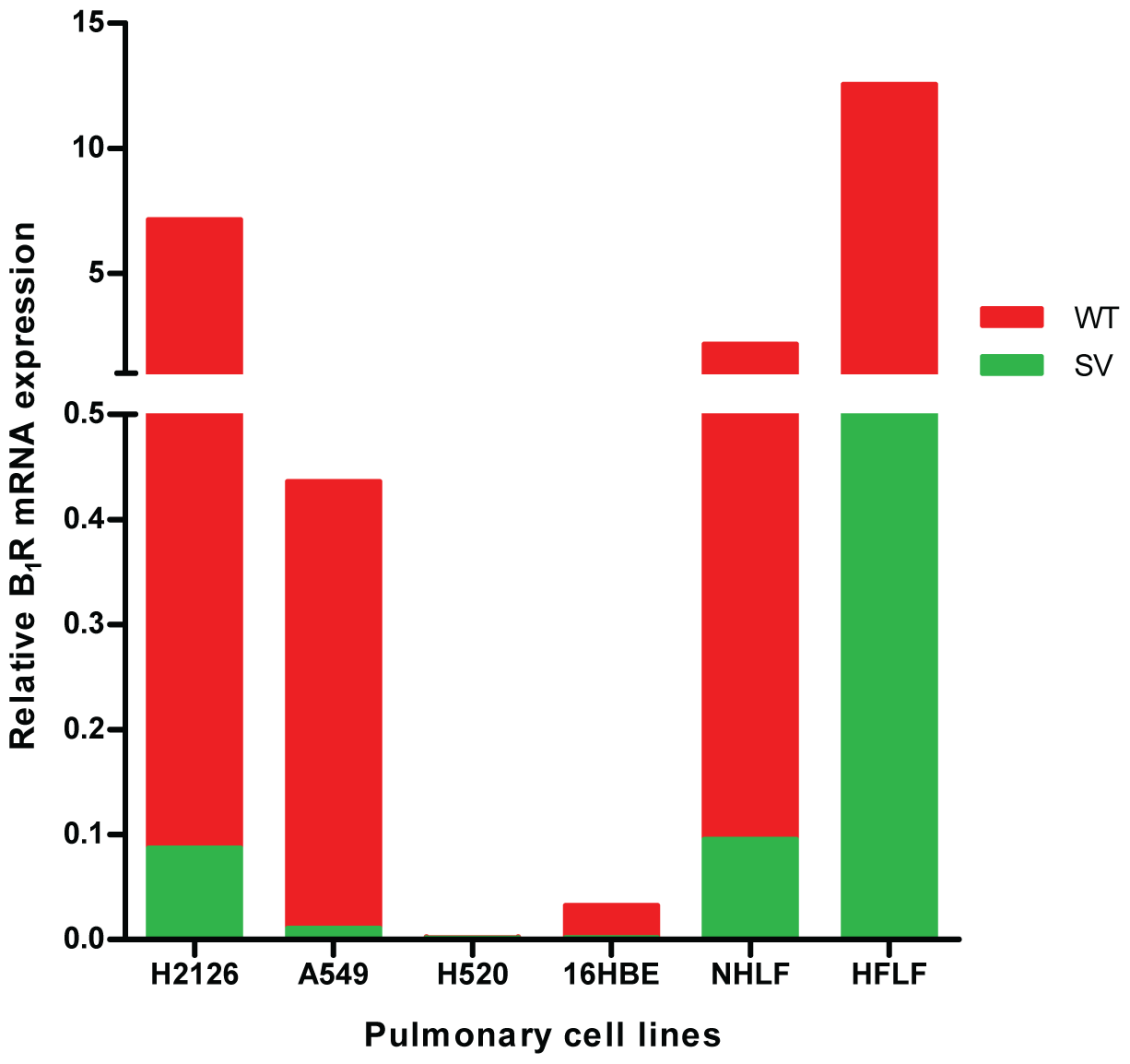
B_1_R transcript is differentially expressed across a range of cell lines. B_1_R mRNA expression was normalised to housekeeping gene SOD1 in human pulmonary cell lines as quantified by real time PCR. Data represents mean ± SEM from 3 independent experiments, each performed in duplicate.

Since the degree of B_1_R expression in airway epithelial and other lung cells is unknown, the basal mRNA expression in six lung cell lines was assessed (Table 1). All cell-lines constitutively expressed B_1_R receptor except for the lung squamous carcinoma cell line H520 (Fig 1). In the 16HBE cell line, expression of B_1_R was very low and we were able to detect it only on using real-time RT PCR. Expression of total B_1_R was the highest in the human fetal lung fibroblast (HFLF) cell line.

### Kinin B_1_ receptor promoter activity in human lung cell lines

We assessed the role of two reported B_1_R promoters and especially the role of cell specific activity in exon II [22] and intron II [22], [23] in regulating B_1_R promoter activity in the high expressing (H2126) and low expressing (16HBE) lung cell-lines. In the high expressing human lung adenocarcinoma H2126 cell-line, the complete promoter with exon 2 and intron 2 construct (CP-E2I2-Luc, p = 0.003) and the promoter alone construct (CP-Luc, p = 0.006), were able to significantly increase luciferase expression compared to pGL3 Basic (data not shown).

**Figure 2:**
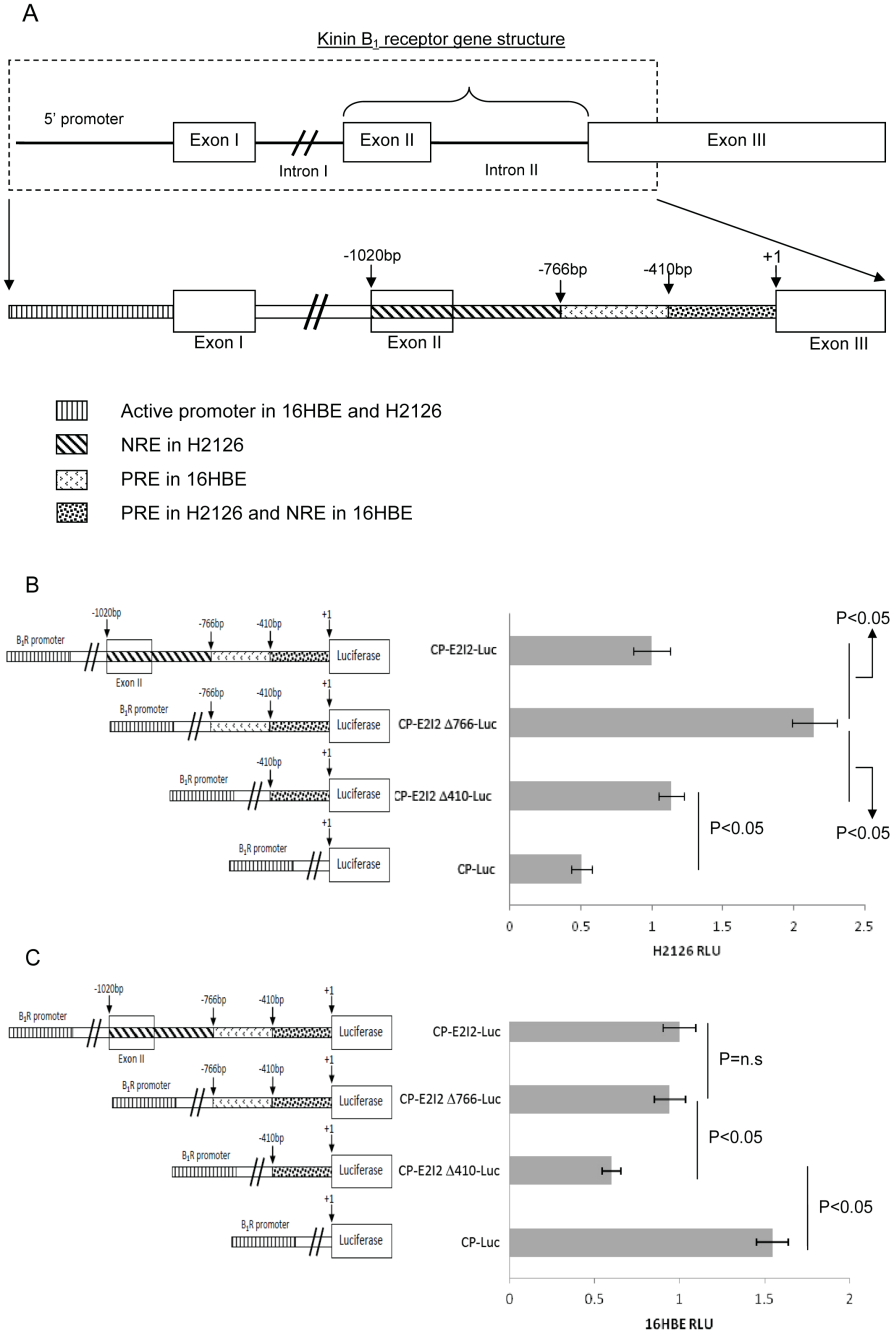
Deletion B_1_R promoter constructs in HIGH expressing H2126 and LOW expressing 16HBE reveal regulatory regions. Summary of results from deletion constructs of B_1_R regulatory regions (A). The size of each regulatory region is indicated relative to start of exon III of B_1_R (+1). PRE = positive regulatory element, NRE =  negative regulatory element. Relative luciferase activity of promoter deletion constructs transfected into human lung adenocarcinoma H2126 (B) and human bronchial epithelium 16HBE (C). Data presented as mean with error bars representing SEM. Data was analysed using one-way ANOVA and Tukey's post-hoc test on 4 independent experiments, each performed using at least triplicates. *p≤0.05 was considered statistically significant. Activity of CP-E2I2-Luc construct containing the B_1_R 5′ promoter and −1020 bp to +1 was set to 1.

However, deletion of −1020 bp to −766 bp in the exon 2-intron 2 interface (CP-E2I2 Δ766-Luc) increased luciferase expression in H2126 by 114% (p = 0.001; Fig. 2B), compared to the complete construct, CP- E2I2-Luc, while deletion of −766 bp to −410 bp (CP-E2I2 Δ410-Luc) did not significantly change promoter activity. The total deletion of the exon 2-intron 2 decreased expression by 55% (p = 0.002; Fig. 2B), compared to the complete promoter with exon 2-intron 2 construct, CP- E2I2-Luc.

In the low expressing human bronchial epithelial cell-line (16HBE), constructs with the complete promoter with exon 2-intron 2 construct (CP- E2I2-Luc, p = 0.003) and the promoter alone construct (CP-Luc, p<0.001) showed significantly increased expression compared to pGL3 Basic (data not shown). While luciferase expression for the promoter alone construct (CP-Luc) was 60% higher (p = 0.004), the exon 2-intron 2 deletion constructs (−1020 bp to −766 bp; CP-E2I2Δ766-Luc) did not significantly alter luciferase expression in 16HBE (Fig. 2C), compared to the complete construct (CP- E2I2-Luc). Additional removal of −766 bp to −410 bp (CP-E2I2 Δ410-Luc) significantly decreased (p = 0.018) luciferase expression compared to CP-E2I2Δ766-Luc. Stimulation of H2126 and 16HBE cells with 100 nM or 1000 nM DAKD for 3, 6 and 24 h did not significantly change the expression level of the promoter constructs (CP- E2I2-Luc, CP-Luc) compared with unstimulated cells (data not shown). Exposure to the general inflammatory stimulus lipopolysaccharide (LPS) 0.1 µg/µL for 3, 6 and 24 h also showed no significant change in luciferase activity (data not shown).

### Multiple transcription start sites of B_1_R and identification of 5′UTR splice variant

The human lung adenocarcinoma (H2126) cell line was selected to determine the TSSs of B_1_R based on its epithelial origin and epithelial-like morphology as well as representing a high B_1_R expressing lung cell-line.

**Figure 3:**
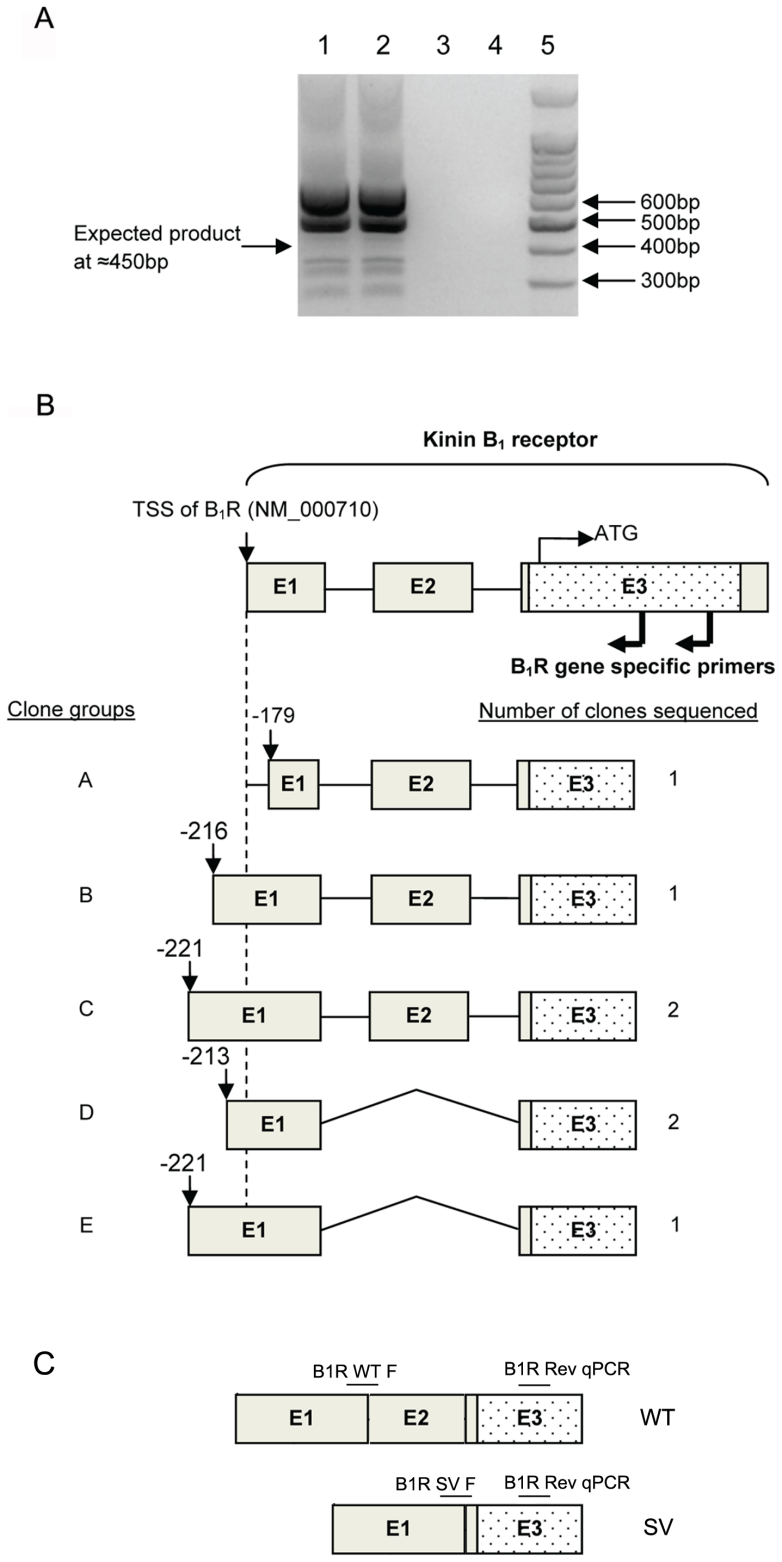
5′RACE PCR analysis of H2126 cDNA reveals multiple products. H2126 cDNA was amplified using the GeneRacer 5′nested primer and RT Rev 2 primer (A). Expected product size was 450 bp although at least 5 other bands were observed. Lanes 1 and 2: H2126 cDNA, Lanes 3 and 4: no template control. **Major transcription start sites (TSS) identified** in this study are labelled relative to translation start site (ATG) of NCBI published sequences of B_1_R (B). TSS of transcript D identified in this study is located 12 bp upstream of TSS on NCBI (NM_000710) but matches TSS identified by Yang & Polgar (1996)[23]. In addition to the full-length wild type B_1_R transcript, a splice variant of B_1_R (transcript D and E) was also identified in this study. The TSS of this splice variant was at two primary locations; 12 bp and 4 bp upstream of NCBI sequence. Schematic presentation of identified wild type (WT) and splice variant (SV) transcripts and position of primers used in RT-qPCR to specifically amplify WT (B1R WT F) and SV (B1R SV F) (C). Forward primers are spanning the splice sites while common reverse primer (B1R Rev qPCR) located in exon 3 was used for amplification of both transcripts.

Based on published B_1_R sequence, a product size of approximately 450 bp was expected. Instead, following 5′ RACE PCR we observed 6 other distinct products (Fig. 3A). These PCR products were cloned and sequenced in order to determine TSS and whether the banding pattern was due to PCR artefacts or if they were due to the presence of alternative B_1_R transcripts in H2126.

Sequence analysis of 7 clones revealed 4 possible TSS in H2126 which separated into 2 major transcript types (Fig. 3B). Transcripts C and E both had TSS corresponding to the sequence published by Yang and Polgar [23] although in their study, only the full length B_1_R transcript was detected (Fig 3B, transcript C). None of them corresponded to the TSS of the published B_1_R mRNA sequence (NM_000710.2). In addition to the alternative start sites, a novel 5′UTR SV which skips exon II of B_1_R was detected (Fig 3B, transcripts D and E).

### Confirmation of novel 5′UTR B_1_R splice variant

**Figure 4:**
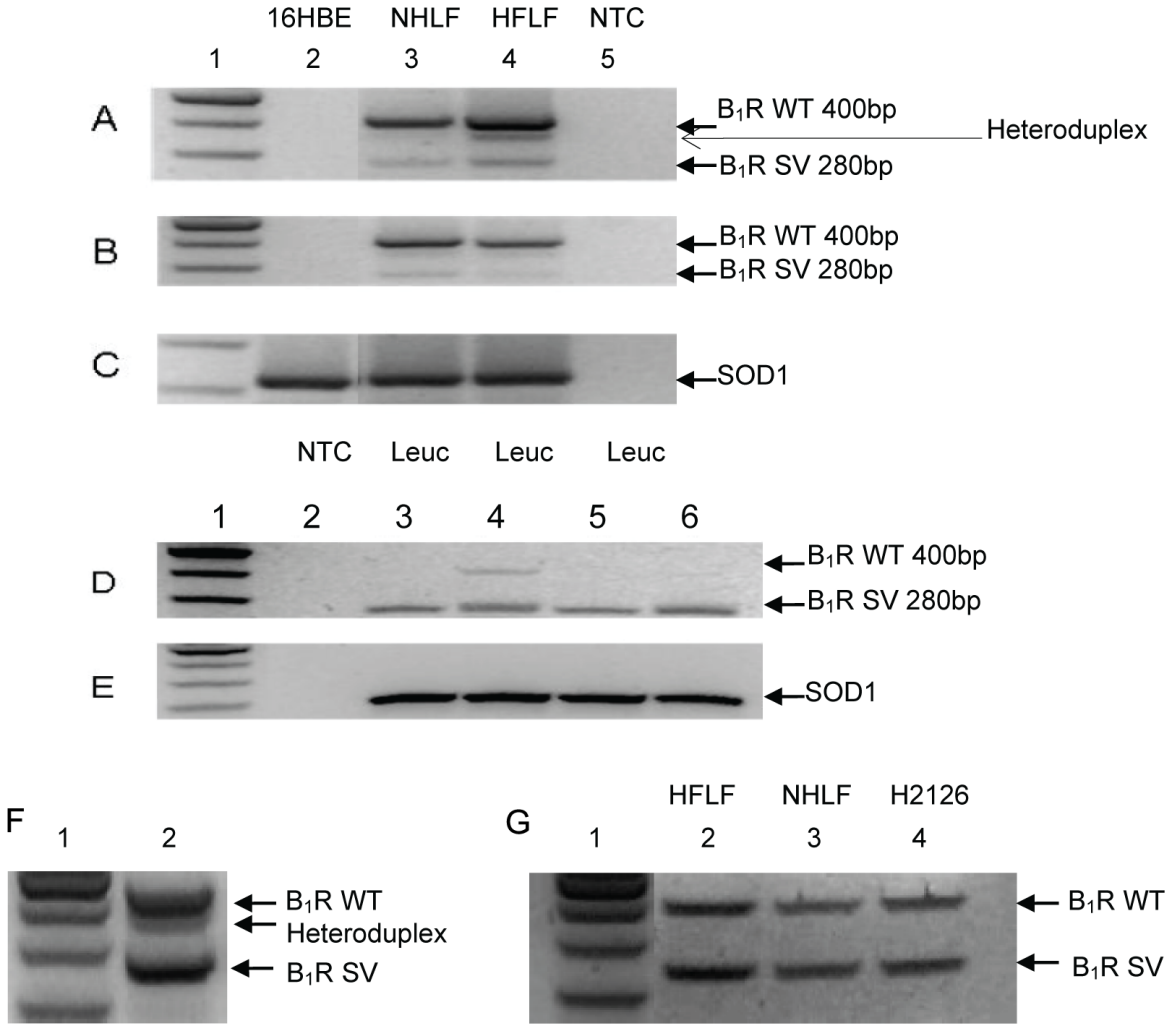
B_1_R splice variant (B_1_R SV) transcript and heteroduplex band is present in several cell types. Amplification of B_1_R WT and SV using primer set RT Fow and RT Rev (A) and RT Fow2A and RT Rev 1 (B). Amplification of loading control housekeeping gene superoxide dismutase 1 (SOD1) (C). Amplification of B_1_R WT and SV from human leucocytes using primers RT Fow and RT Rev (D) and amplification of SOD1 (E). A representative image of additional heteroduplex band located between the WT and SV band in HFLF. Three extra PCR cycles were used to match conditions used in G, but without addition of 10X fresh PCR mix (F). Heteroduplex band removed after addition of 10X fresh PCR mix followed by 3 PCR cycles (G). Lane 1: 100 bp ladder. PCR no template control (NTC). Human leucocytes (Leuc).

B_1_R SV was detected using two different sets of common primers located in exon I (Forward primers: B_1_R Fow or B_1_R Fow 2A) and exon III (Reverse primers: B_1_R Rev or B_1_R Rev1) (Fig. 4A, Table 2). The result of each primer set showed an additional band 120bp smaller than the expected wild-type B_1_R product indicating presence of B_1_R SV (Fig. 4A). Sequencing of the B_1_R SV band confirmed the identification of the novel B_1_R transcript that splices out exon II exactly at the intron/exon boundary.

To determine if alternative splicing was an artefact of the immortalised cell line, H2126, RNA from other human pulmonary cell lines (16HBE, HFLF and NHLF) along with human leucocytes were used to determine the presence of the B_1_R 5′UTR B_1_R SV. The B_1_R SV was easily detected in lung fibroblasts (HFLF, NHLF) and human leucocytes but not bronchial epithelial cells (16HBE) (Fig. 4A). Where the B_1_R SV could be detected, the spliced transcript was less intense than the wild-type/full length (B_1_R WT) transcript except in leucocytes, where the opposite was observed (Fig. 4A).

An additional product which was suspected to be a heteroduplex PCR product was also detected between WT and SV transcripts (Fig. 4A). Studies have shown that transcripts with similar nucleotide regions may form heteroduplexes during PCR [25], [26]. To test whether this band was a heteroduplex, a method used by Thompson *et al* [25] was followed where the first PCR reaction was diluted 10X with fresh PCR mix and the reaction was run for 3 cycles of denaturation, annealing and extension at conditions used in the previous PCR. Using this method, heteroduplexes are unable to form due to an excess of PCR mix components. This demonstrated that the product in question was indeed a heteroduplex and this result was reproducible in different cell types (Fig. 4G).

### Differential cell expression of the novel 5′UTR B_1_R splice variant

Quantitation of B_1_R WT and SV transcript expression in a range of human pulmonary cell lines was tested. Results confirmed the qualitative results mentioned previously, with the SV detected in significant amounts in lung fibroblasts (HFLF, NHLF) and lung adenocarcinoma cell-lines (A549, H2126) (Fig. 1). However, the spliced transcript was expressed in significantly less amounts than the wild-type/full length (B_1_R WT) transcript (Fig. 1).

### B_1_R WT mRNA is more stable than 5′UTR SV mRNA

**Figure 5:**
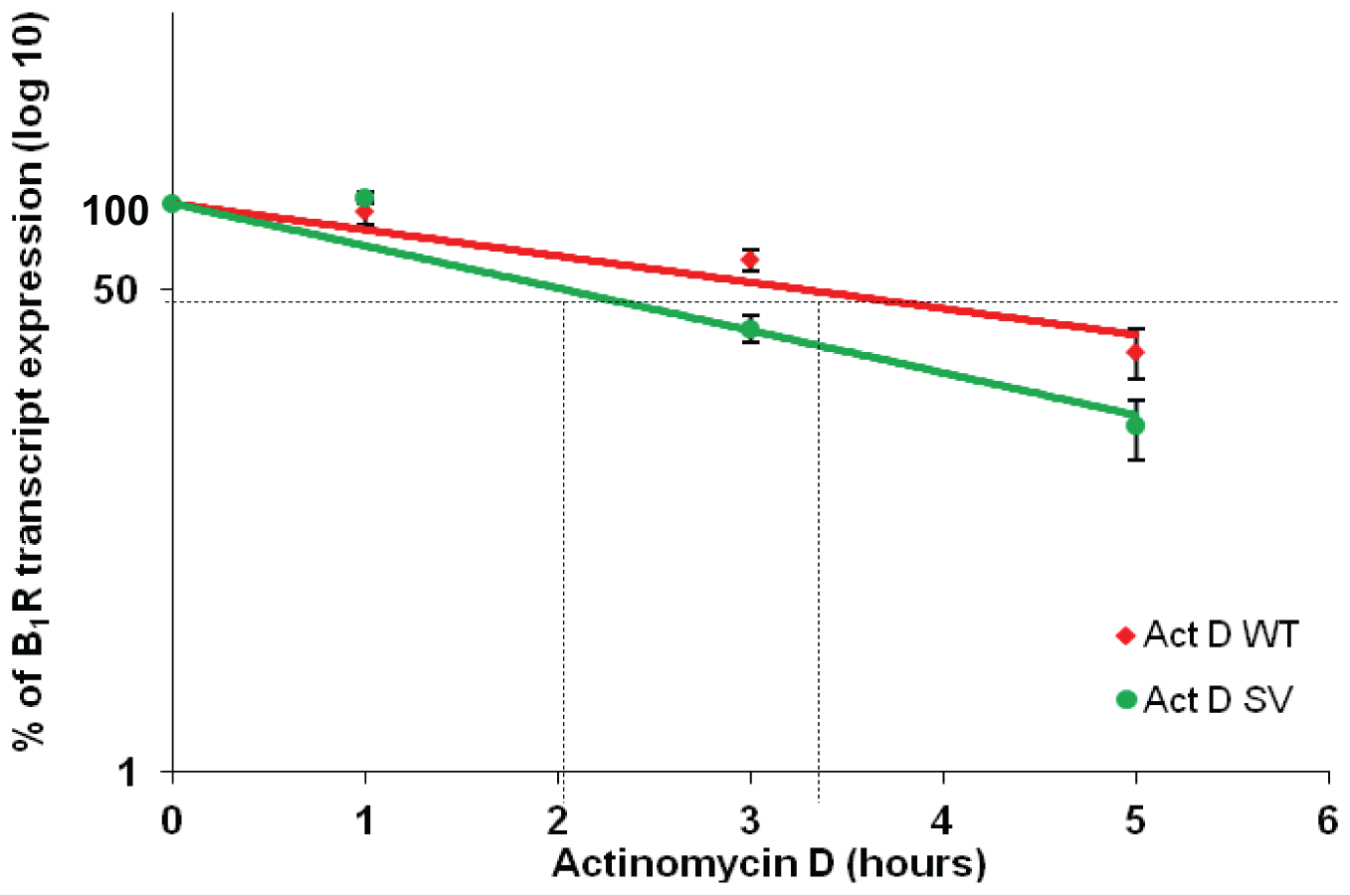
B_1_R WT is more stable than B_1_R SV under basal conditions. Actinomycin D (Act D) mRNA decay of B_1_R WT and SV transcripts in H2126 measured at 0, 1, 3, 5h using real time PCR (Act D treatment at concentration of 5 µg/mL). Data plotted is mean±SEM from four independent experiments each performed at least in duplicates. Half-life of mRNA can be roughly estimated by determining the time required to reach 50% transcript level (shown by dotted lines). For more accurate assessment, the trendline equations obtained by plotting the graph are used to determine the half-life. In this graph, the equation for B_1_R WT is y = 100e^−0.213x^ while for B_1_R SV is y = 100e^−0.344x^, where y is set to 50 (indicating 50% of transcript remaining) which will allow the calculation of x (indicating time required to reach 50% transcript level). From these equations, the half-life of B_1_R WT is 3.28 hr and 2.02 hr for B_1_R SV.

Sequencing results confirmed that the 5′UTR B_1_R splice variant is 120 bp shorter than the WT. Variations in the 5′ UTR can affect the stability of mRNA and the efficiency of protein production. To determine the stability of WT and SV B_1_R mRNA, the mRNA half-life was determined up to 5 hr after incubation of H2126 cells with a transcription inhibitor, actinomycin D (Act D). The calculated half-life of the WT and SV transcripts was 3.28 and 2.02 hr, respectively (Fig. 5; t-test, p = 0.04).

### B_1_R 5′UTR SV transcript does not affect translational efficiency

**Figure 6:**
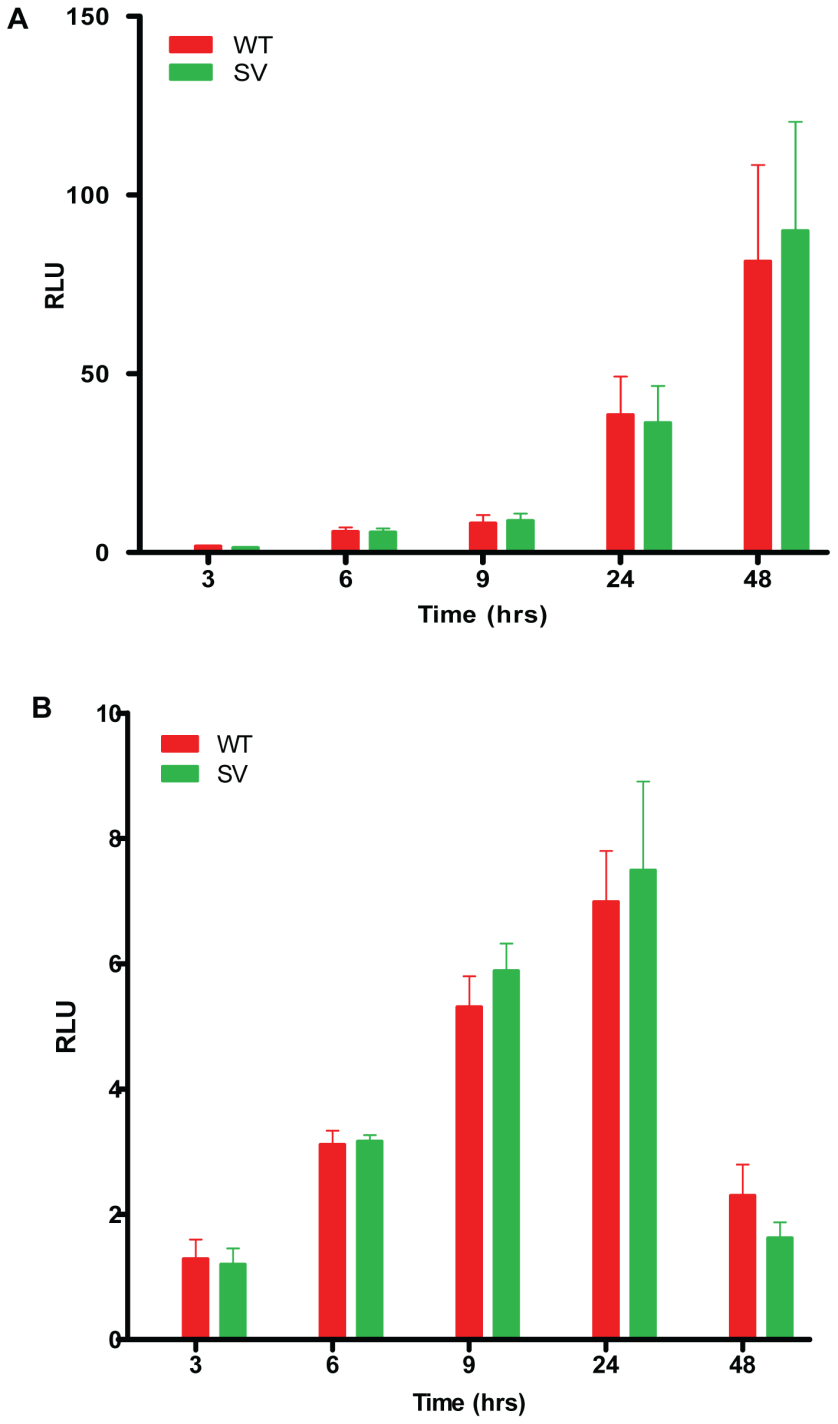
B_1_R SV does not affect translation efficiency. Translation efficiency of B_1_R WT and SV 5′ UTR measured using luciferase expression normalised to Renilla expression over time. Transfection of WT-luciferase and SV-luciferase constructs into normal lung bronchial epithelial, 16HBE (A), and lung adenocarcinoma, H2126 (B). Results are the average of five experiments with error bars representing SEM. There was no significant difference between the translation efficiency of B_1_R WT and B_1_R SV.

The 5′ UTR can also play an important role in regulating the rate of mRNA translation to protein. To determine if the SV transcript affects the rate at which B_1_R protein is produced, the WT and 5′ UTR SV were inserted immediately upstream of the luciferase coding region and immediately downstream of a SV40 promoter. 16HBE and H2126 cells were then transfected with the constructs and luciferase activity measured over 48 hr. There was no significant difference in the rate of protein produced using either UTR construct (Fig. 6). However, there was a noted difference in the overall pattern of luciferase expression depending on cell type. In 16HBE the luciferase protein increased gradually peaking at 48 hrs, while in H2126 it peaked at 24 hr before falling almost to basal levels by 48 hr (Fig. 6).

### Effect of B_1_R specific stimulant DAKD on B_1_R WT and SV mRNA expression

**Figure 7:**
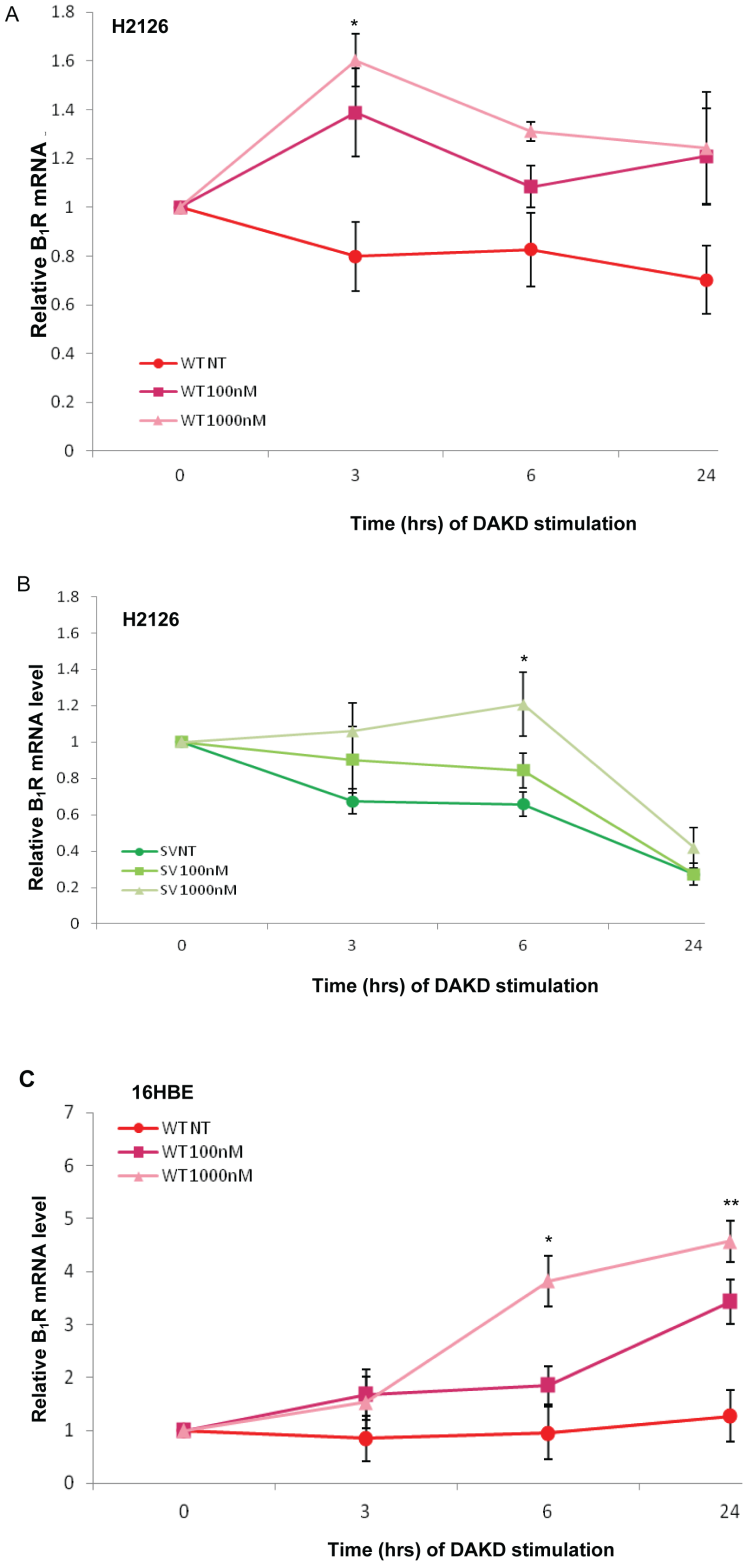
B_1_R WT and SV expression following DAKD stimulation. Quantitative real-time PCR measurements of time (0, 3, 6 and 24 hr) and dose effect of DAKD (100 nM and 1000 nM) on B_1_R WT (A) and SV (B) expression in H2126 and on B_1_R WT expression in 16HBE (C). B_1_R mRNA level at 0 hr was set to 1. Data from 4 experiments performed in duplicates with mean ± SEM represented. DAKD treated samples were compared with non-treated, media only (NT) for each time point. Data was analysed using unpaired Student's t-test where *p<0.05 is considered statistically significant. **p<0.001

B_1_R is an inducible gene upregulated by several inflammatory stimuli including its agonist DAKD. To determine if the WT and SV transcripts are differentially affected by DAKD, H2126 and 16HBE cells were incubated with 100 nM and 1000 nM of DAKD over a 24 hr period. B_1_R WT and SV gene expression (Fig. 7A & B) showed that in H2126 the WT and SV transcripts were significantly induced with 1000 nM DAKD but at different times with a peak at 3 hr for WT (increased 60%, p = 0.03) and 6 hr for SV (increased 25%, p = 0.04). In 16HBE cells WT, but not SV, mRNA expression was increased following 1000 nM DAKD stimulation for 6 hr 250%, p = 0.03) and remained elevated until 24 hr (300%, p = 0.008;Fig. 7C).

## Discussion

Although kinins play an important role in airway inflammation, the regulation of the inflammation-induced kinin receptor B_1_R expression in pulmonary cells is unknown. We identified a novel B_1_R SV which is less stable than wild-type mRNA but does not appear to impact on translation efficiency. The differential constitutive and stimulated expression of SV compared to wild-type B_1_R suggests a role of SV in regulating B_1_R gene expression in human airway cells. In addition, we have identified regulatory elements, rather than a previously proposed alternative promoter, in exon II and intron II of the 5′UTR regulating the expression of B_1_R. These findings reveal complex regulation of B_1_R receptor expression which may enable its future manipulation in chronic pulmonary inflammation and cancer.

B_1_R is expressed by a range of cells and tissues including the lung and is rapidly induced during inflammation [4]–[7]. Our group and others have reported constitutive B_1_R expression in neutrophils [27], primary sensory A- and C-fibers [28], eosinophils [17], macrophages [29], dendritic cells [30], [31], and pulmonary primary cells [9], [10] and cell-lines [10], [32], [33]. In agreement with our previous work we found higher constitutive expression of B_1_R in pulmonary adenocarcinoma cells (H2126, A549) and pulmonary fibroblasts (NHLF, HFLF) compared to normal bronchial epithelial cells (16HBE), while squamous cell carcinoma cells (H520) did not constitutively express B_1_R [34].

Following DAKD stimulation of one of the high constitutive expressors (H2126) and low constitutive expressors (16HBE) we found that the low constitutive expressor was more responsive to DAKD compared to the high constitutive expressor. Previous studies have also show high B_1_R constitutive expression in a number of different cancers, including lung [35]. Further, B_1_R antagonists have been efficient in inhibiting growth in a range of lung cancers NSCLC, SCLC and mesothelioma [36], [37]. In contrast, low constitutive B_1_R expression is upregulated in human nasal epithelial cells in allergic rhinitis subjects compared to controls [9] and in human primary bronchial epithelial cells post stimulation with IL-1β and TNF-α [10]. These differences may be explained by cell-specific regulatory mechanisms which we subsequently investigated.

Using lung fibroblast and smooth-muscle cells others have reported B_1_R core and alternative promoters with the 5′ core promoter defined as 1.4 kb upstream of exon I and the alternative promoter 1020 bp upstream of exon III (intron II and exon II)[21], [22]. However, there is no published evidence of a TSS driving this alternative promoter. We identified similar regulatory elements in the 5′UTR in two pulmonary cell-lines (Fig 2). The −1020 to −766 bp region of 5′UTR acts as a NRE only in high expressing H2126 cells with no effect in low expressing 16HBE. In contrast, enhancer-like elements between −1842 and −812 were previously reported in HepG2 cells [22], suggesting that this region may be responsible for cell specific activity. While this group found no further regulatory elements downstream we identified an enhancer-like element between −766 and −410 bp in the low expressing 16HBE and found that the region −410 to +1 acts as a PRE in H2126 cells and in contrast as a NRE in 16HBE cells. We demonstrated that −400 to +1 bp contains the minimum sequence required for promoter activity supported by others that report 300 bp as the minimal region [22]. We also noted that in the constructs without core promoter (data not shown), basal activity of −410 to +1 region is higher in high expressor cells than in 16-HBE low expressor cells suggesting this region may be involved in constitutive, cell-specific receptor expression.

To determine whether the 5′UTR regulatory regions could affect induction of B_1_R, 16HBE and H2126 cells were stimulated with pro-inflammatory LPS and DAKD. Neither stimulus affected luciferase activity of any promoter construct. The lack of induction by LPS is in agreement with previous studies showing that LPS as well as IL-1β and TNF-α do not induce activity in human HepG2 and rat vascular smooth muscle cells [18]. The inability of DAKD to induce activity is not surprising as no consensus has been achieved from previous studies [18], [19], [24], [38], [39]. The majority of studies have failed to induce 5′ core promoter activity in human lung fibroblasts, human smooth muscle cells and peripheral blood lymphocytes [19], [21], [38]. This highlights the tight and delicate balance of B_1_R regulation at the promoter level and is an indication that other regions outside of the promoter, exon II and intron II of B_1_R, are more likely to play a role in the upregulation of B_1_R by LPS and DAKD. In an attempt to locate the domains involved, Yang *et al* [38] constructed a human B_1_R minigene that consisted of 1.8 kb of the promoter, exon I, 1.5 kb of intron I, exon 2, intron 2 and luciferase gene. This minigene exhibited promoter activity with LPS and DAKD stimulation, which was abolished with the replacement of the minigene with 1.8 kb 5′ promoter construct.

To investigate whether these regulatory regions acted as a promoter in pulmonary cells we looked for alternative TSS. While we could not detect any TSS downstream of the proposed alternative promoter, we cannot exclude that this region does not act as a promoter in other cell types or in developmental stages as intronic promoters can regulate transcripts which are tissue- or differentiation-specific [40]. However, we did identify a novel kinin B_1_R SV, with exon II skipping, which adheres to the consensus GT-AG sequence conserved in 98% of mammalian splice sites [41], [42]. This SV was detectable in a range of human immortalised pulmonary cell lines, as well as primary human leucocytes and lung tissue (data not shown). The splice variant was the dominant transcript in human leucocytes in contrast to pulmonary cell-lines.

Exon II splicing of B_1_R has not been documented in any other species. However, several SVs in 5′UTR have been reported in rat B_1_R including a 41 bp skipping at the start of the exon II which was predicted to affect translation efficiency [43]–[45]. Results from these and our study, suggest that 5′UTR splicing may be a common event in the regulation of kinin receptors.

This novel human SV affects only the 5′UTR of B_1_R while the coding region and protein remain unchanged. 5′ untranslated regions regulate the efficiency of protein translation as well as the stability of the transcript [46]. The WT transcript half-life measured in this study was approximately 108 min and 48 min longer than the results obtained from Zhou *et al* and Schanstra *et al* respectively, who measured the half-life in human embryonic lung fibroblasts-IMR90 [7], [24]. In H2126 cells, the B_1_R SV was ≈ 35% less stable than the wild-type B_1_R which may indicate a possible stabilizing element located within exon II. Characterisation of the gene structure of the human B_1_R suggests that exon II is part of an Alu-J element that spans part of intron I, exon II and part of intron II [23]. Alu elements are small interspersed nucleotide elements which affect gene expression by influencing initiation of transcription and alternative splicing [47], [48], initiation of translation, and translation efficiency [49]–[52]. More recently, the presence of Alu elements in exons and adjacent introns has been linked to forming circular RNAs, which have increasingly been reported as strong regulators of gene expression [53]. In particular, circular RNAs are cell- and developmental stage-specific post-transcriptional regulators which compete for binding by microRNAs or RNA binding proteins [54] and may contribute to the cell-specific differences in receptor expression we observed.

Our *in silico* analysis of B_1_R mRNA folding and secondary structure predicts that the B_1_R wild-type 5′ UTR is more stable with a free energy of −60.30 kcal/mol compared to B_1_R splice-variant at −16.10 kcal/mol. No discernible difference between the wild-type and SV UTR translational efficiency was observed suggesting the 5′UTR is not involved in B_1_R translational efficiency. At all earlier time points including 9 hr luciferase expression was the same between both cells lines. At 24 and 48 hr there was up to a 10 fold higher luciferase expression in the lower constitutive expressing 16HBE compared to the high constitutive expressing adenocarcinoma H2126. The difference in rate of translation between 16HBE and H2126 may reflect a more active SV40 promoter in 16-HBE or the lack of B_1_R 3′UTR down regulatory elements in these constructs.

Cell specific differences in WT and SV expression were observed post stimulation. While in both cell lines B_1_R SVs were inducible, delayed upregulation of the SV transcript in H2126 cells following stimulation with the B_1_R-specific agonist DAKD suggests that the SV is regulated in a different manner to the WT. The profile of total B_1_R mRNA expression in H2126 correlates well with other studies that indicate an increase in mRNA 2–3 hr post-stimulation which is maintained at 4–6 hr and falls by 12 hr [55], [56]. In 16HBE cells, the WT mRNA expression post-stimulation was highest at 24 hr. As mentioned earlier, B_1_R SV expression in 16HBE was undetectable. This low expression of B_1_R SV suggests that the SV may not be essential to the regulation of B_1_R expression in 16HBE. The time-dependent increase in B_1_R mRNA transcripts following DAKD stimulation may be due to either increased mRNA production and/or increased accumulation of mRNA due to less degradation/increased stability. The transcriptional regulatory effect of DAKD on B_1_R is mainly through NF-kB and AP-1 [18], [24], [57], [58]. As both B_1_R transcripts arise from transcription initiated from the same TSS, it is unlikely that DAKD stimulation increases specific B_1_R transcripts through promoter regulation. Increasing stability of SV transcript could be a plausible mechanism whereby DAKD, either directly or indirectly, stabilizes the mRNA leading to accumulation of the SV we describe.

## Conclusions

This study has identified the existence of a novel and naturally occurring SV of human B_1_R that reduces the length of the 5′UTR region of B_1_R. Characterisation of the effect of 5′UTR in terms of mRNA stability and translation efficiency revealed that the novel SV is 35% less stable than the wild-type full length transcript in H2126 cells but does not impact on the translation efficiency of the downstream protein as measured by luciferase activity. The DAKD agonist differentially increased B_1_R mRNA WT and SV that may be important in maintaining a more chronic response during disease. The importance of the identified regulatory elements present in the B_1_R 5′UTR and the alternative splicing with the possibility of forming new classes of regulatory RNAs and influence on the cell specific expression needs to be further investigated. It can be speculated from these studies that cells expressing high constitutive levels of B_1_R, reported to occur in disease states, are regulated by the production of a exon II splicing SV and cell specific regulatory elements within the ‘alternative promoter’. While more research is required to elucidate this observation, our findings suggest that specific targets may be available to downregulate B_1_R expression in inflammatory diseases in particular asthma, COPD and cancer.
